# A Comprehensive, Simple, Robust, and Solvent-Free Method Covering Ultrashort- to Long-Chain PFAS in Atmospheric Samples

**DOI:** 10.1021/acs.analchem.5c03123

**Published:** 2025-07-02

**Authors:** Wanlin Guo, Yanhao Zhang, Yawei Wang, Lin Zhu, Zongwei Cai

**Affiliations:** † State Key Laboratory of Environmental and Biological Analysis, Department of Chemistry, 26679 Hong Kong Baptist University, Kowloon, Hong Kong SAR 999077, China; ‡ School of Ecology and Environment, Zhengzhou University, Zhengzhou 450001, China; § State Key Laboratory of Environmental Chemistry and Ecotoxicology, 12381 Research Center for Eco-Environmental Sciences, Chinese Academy of Sciences, Beijing 100085, China; ∥ Eastern Institute of Technology, Ningbo 315200, China

## Abstract

Ultrashort- and short-chain perfluoroalkyl and polyfluoroalkyl substances (PFAS), including trifluoroacetic acid (TFA), are emerging as widespread and persistent atmospheric pollutants of growing concern. Their atmospheric accumulation is further exacerbated by the transformation from various precursors, such as long-chain perfluorocarboxylic acids (PFCAs) and neutral PFAS. An efficient analytical method covering ultrashort- to long-chain PFAS is therefore required to monitor environmental levels and understand transformation mechanisms. However, distinct polarity among these PFAS poses technical challenges for simultaneous detection within a single run, hindering the comprehensive understanding of degradation mechanisms and quantitative correlation analysis. Conventional methods using liquid chromatography-electrospray ionization (LC-ESI) are effective for medium- to long-chain PFAS but are limited in detecting ultrashort-chain species concurrently. Herein, we present a simple yet robust method for broad-spectrum PFAS analysis, covering ultrashort- to long-chain species, using dielectric barrier discharge ionization (DBDI) coupled directly to high-resolution tandem mass spectrometry (HRMS/MS). This approach enables efficient ionization across a wide polarity range with reduced intensity of in-source fragmentation (ISF). Moreover, solid-phase microextraction (SPME) simplifies labor- and time-intensive sample preparation without solvents. As a result, a high sensitivity of 0.06–2.02 pg/m^3^ was achieved with minimal background interference, and ISF was reduced by over 60% compared to existing methods. Using this approach, we explored potential environmental associations between PFAS and cooccurring pollutants in seasonal atmospheric samples, showcasing its utility for future environmental research.

## Graphical abstract

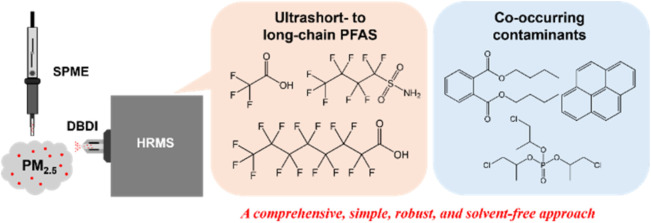

## Introduction

Polyfluoroalkyl substances (PFAS), a large family comprising over 10,000 compounds, were widely used in modern industry.^1,2^ There has been an emerging environmental presence of short-chain and ultrashort-chain PFAS as substitutes for their long-chain counterparts.^3–5^ The rising accumulation of these short-chain PFAS poses significant ecological and human health risks.^6–10^ In addition, environmental accumulation of short-chain PFAS, particularly 2-carbon TFA, is found to be underestimated recently, arguably due to the atmospheric transformation from various precursors such as long-chain PFCAs and neutral PFAS.^11–13^ The complex transformation pathways of PFAS, involving processes like photodegradation and oxidation, complicate source attribution and highlight the urgent need for comprehensive monitoring of both short-chain PFAS and their long-chain precursors.^11,14–16^ However, the study with simultaneous measurement of volatile PFAS and neutral precursors is limited, primarily due to low atmospheric concentrations and lack of efficient analytic tools.^17,18^

Capabilities for such comprehensive analysis covering short-chain and long-chain PFAS compounds with diverse volatility and polarity are still limited.^9,19,20^ While gas chromatography (GC)-MS could be used for TFA analysis, it is limited in both sensitivity due to derivatization requirements and analytical capabilities to cover longer-chain species.^16,21^ LC-ESI-MS has emerged as the gold standard for sensitive detection of medium- to long-chain PFAS at a trace level, as demonstrated by the study of Zheng et al., which enabled high-throughput analysis of 56 PFAS species.^22^ However, it faces significant challenges in detecting highly polar and hydrophilic ultrashort-chain PFAS, particularly TFA, which requires specialized separation techniques.^23,24^ The feasibility of rapid, high-throughput analysis of PFAS in water using microfluidic ESI has been recently demonstrated but limited to compounds with chain length >4 carbons.^25^ To date, there is only one study that achieved quantification of ambient ultrashort- and short-chain PFCAs alongside their neutral precursors using LC-ESI-MS.^26^ Nevertheless, different separation conditions are required for ultrashort-chain PFAS and other PFAS, making it laborious and complex in the analytical procedure.

Furthermore, the inevitable ISF phenomenon presents an inherent challenge in ESI of PFAS by reducing selectivity and sensitivity, particularly with elevated source temperatures and fragmentor voltages.^27,28^ A recent study has demonstrated 100% ISF occurrence rates in six out of eight PFAS classes analyzed by LC-ESI-MS, with pronounced ratios observed for short-chain congeners due to their characteristically lower bond dissociation energies.^28^ Moreover, the pervasive PFAS contamination in laboratory solvents and consumables necessitates stringent blank control and specialized low-background materials to ensure accurate trace-level quantification.^29^

DBDI, an advanced ambient ionization mass spectrometry (AIMS) technique, exhibits exceptional ionization efficiency across a broad range of polarities.^30–32^ The diverse yet soft ionization process might be suitable for comprehensive profiling of a broad spectrum of PFAS without significant ISF, yet it has not been confirmed.^33,34^ In addition, current AIMS approaches relying solely on full-scan mode suffer from isobaric interferences and matrix effects; thus, we incorporated MS/MS mode to enhance analytical specificity.^31,35,36^

In this study, we integrated SPME with AIMS for a comprehensive, simple, and robust analytic strategy of PFAS covering ultrashort- to long-chain length, with significantly less ISF. Fine particulate matter (PM_2.5_) samples,^37–41^ complex chemical matrix carrying a wide range of airborne pollutants, were used as a proof of concept. Our method provides a novel tool for exploring the potential environmental association between ultrashort-chain PFAS with their precursors and coexisting pollutants in the seasonal PM_2.5_ samples, enabling future environmental investigations.

## Materials and Methods

### Chemicals and Materials

Chemical standards, including TFA (99%), polycyclic aromatic hydrocarbons (PAHs), organophosphate esters (OPEs), and phthalate esters (PAEs), pyrene-*d*_10_ (Pyr-*d*_10_), diethyl phthalate-*d*_4_ (DEP-*d*_4_), and tris­(2-chloroethyl) phosphate-*d*_12_ (TCEP-*d*_12_), along with four types of SPME fibers, were purchased from Sigma-Aldrich (St. Louis, MO). Additional PFAS solutions (10 μg/mL) were obtained from Alta Scientific (Tianjin, China), encompassing ultrashort-chain (C2–C3), short-chain (C4–C6), and long-chain (C8) PFCA (C*_n_* F_2*n*+1_COOH); short-chain (C4, C6) and long-chain (C8) perfluoroalkyl sulfonamides (FASAs, C*_n_* F_2*n*+1_SO_2_NH_2_); hexafluoropropylene oxide-dimer acid (GenX, C6); *N*-methylperfluorooctanesulfonamide (MeFOSA); 2-(*N*-ethylperfluorooctanesulfonamido) acetic acid (EtFOSAA); and ^13^C_8_-PFOA. The chemical names and structures of all compounds are provided in Figures 1 and S1.

**Figure 1:**
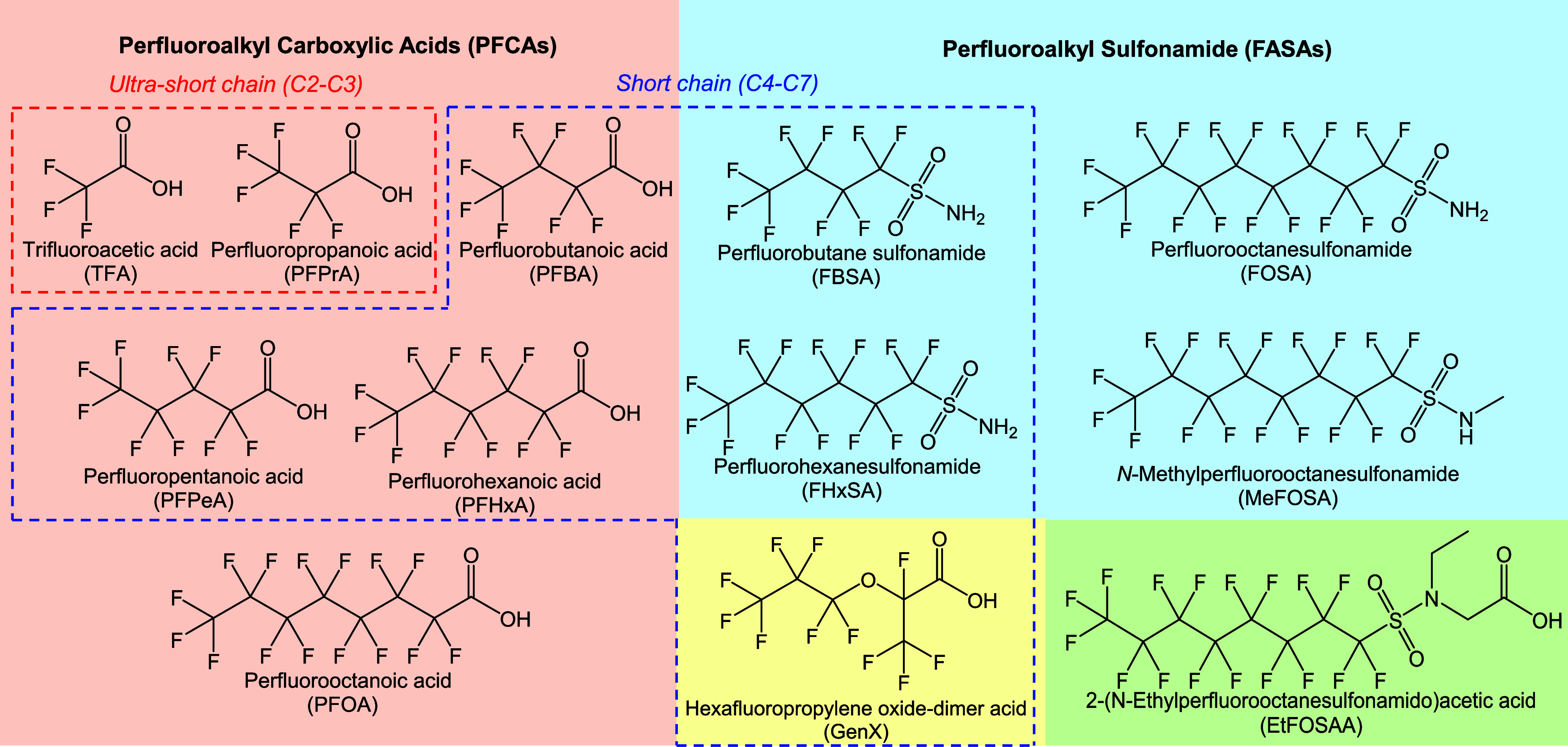
Structural information on subclasses of PFAS. TFA and PFPrA are characterized as ultrashort-chain PFAS (C2–C3); PFBA, PFPeA, PFHxA, FBSA, FHxSA, and GenX are characterized as short-chain PFAS (C4–C7).

### PM_2.5_ Sample Collection

PM_2.5_ samples were collected at Zhengzhou University, China (34°49′ N, 113°32′ E), from December 2023 to August 2024. Sampling dates are listed in Table S1. A TH-1000C high-volume air sampler (Wuhan Tianhong Environmental Protection Industry Co., Ltd., China) equipped with 203 × 254 mm^2^ quartz fiber filters was used, operating at a flow rate of 1.05 m^3^/min for 23.5 h per sample. A total of 24 PM_2.5_ samples were collected, immediately transported to the laboratory, and stored at −20 °C until analysis.

### SPME Optimization and Sample Preparation

SPME conditions were optimized using a standard mixture (1 μg/mL) containing different groups of analytes, spiked onto one-eighth of a blank filter. After air-drying, the filter section was cut into strips and transferred to a 20 mL headspace vial. Four SPME fiber coatings were evaluated: 50/30 μm divinylbenzene/carboxen/poly­(dimethylsiloxane) (DVB/CAR/PDMS), 100 μm poly­(dimethylsiloxane) (PDMS), 65 μm divinylbenzene/poly­(dimethylsiloxane) (DVB/PDMS), and 85 μm polyacrylate (PA). Extraction temperature (40–80 °C) and duration (10–60 min) were systematically optimized.

For sample analysis, one-eighth sections of PM_2.5_ filters were excised using clean tools and placed into 20 mL amber headspace vials. Prior to SPME, a mixture of isotopically labeled internal standards (^13^C_8_-PFOA, Pyr-*d*_10_, DEP-*d*_4_, and TCEP-*d*_12_) at 1 μg/mL was spiked onto each filter section.

### Instrumental Analysis

Multiclass analyte screening was performed using a commercially available DBDI technique, soft chemical ionization in transfer (SICRIT) SC-10 (Plasmion GmbH, Augsburg, Germany) coupled to a high-resolution mass spectrometer, Revident LC/Q-TOF (Agilent Technologies, Santa Clara). The DBDI source was operated at 1.6 and 15 kHz. As illustrated in Figure 2, analyte desorption from SPME fibers was conducted manually using a custom-built SPME holder integrated with a GC/SPME module and a glass inlet liner (Plasmion GmbH, Augsburg, Germany). Desorption duration was set to 2 min following optimization of the desorption temperature (210–250 °C).

**Figure 2:**
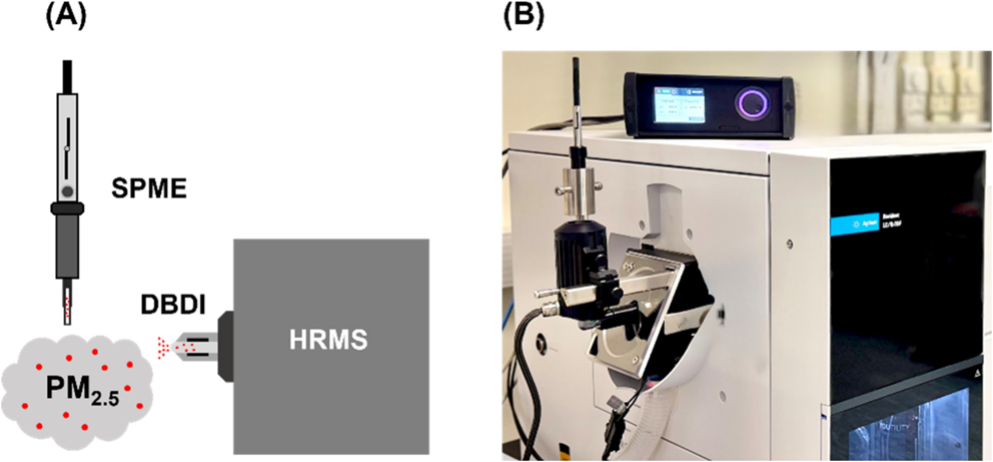
(A) Schematic illustration and (B) photographic image of the instrument setup.

Full-scan MS spectra of individual compound standards were acquired in negative ion mode for PFAS optimization and in positive ion mode for other compound classes. In-source fragmentation was investigated by varying the fragmentor voltage (50–300 V). Additional MS parameters for positive and negative ionization modes, including skimmer and capillary voltages and source temperatures, were optimized and summarized in Table S2. Ionization efficiency and fragmentation were evaluated using air, dry nitrogen, and humidified nitrogen as reagent gases. Mass calibration was conducted using characteristic background ions generated by DBDI, achieving a mass detection accuracy of <5 ppm. In negative ion mode, calibration was performed using nitrate-related reagent ions at *m*/*z* 61.9884 ([NO_3_]^−^) and 124.9840 ([HNO_3_NO_3_]^−^), while positive ion mode calibration utilized background ions at *m*/*z* 149.0233 ([C_8_H_5_O_3_]^+^) and 279.159091 ([C_16_H_23_O_4_]^+^).

Targeted MS/MS experiments, employed for the establishment of an in-house database, were conducted using collision-induced dissociation (CID) with nitrogen as the collision gas. Collision energies ranging from 10 to 40 eV were optimized for each target analyte. An in-house database was established comprising accurate masses and characteristic fragment ions for all target analytes.

### Identification, Quantification, and Data Analysis

High-quality MS/MS spectra were acquired for each chemical standard (PFAS and other target pollutants) under optimized CID conditions. These spectra were then imported into MassHunter Qualitative Analysis 12.0 (Agilent Technologies, Santa Clara) to construct a custom database. For each compound, the most abundant and characteristic product ions were selected for targeted screening, providing the basis of a pseudomultiple reaction monitoring (pseudo-MRM) method. Accurate mass measurements from Q-TOF, corroborated by publicly available databases (Massbank, PubChem) and “in silico” fragmentation tools as needed, were used to confirm the proposed formulas of the selected fragment ions. Quantitative product ions were extracted from the MS/MS data using extracted ion chromatograms (EICs) with a 5 ppm mass tolerance window.

Quantification was performed by using matrix-matched calibration curves for each analyte. Isotopically labeled internal standards were corrected for instrument response and sample preparation variability. Calibration standards (0–100 ng/g) were analyzed by using the optimized DBDI-HRMS/MS method. Calibration curves were generated by plotting peak area ratios of target analytes to their respective internal standards against the concentration ratios. Linearity was assessed using the coefficient of determination (*R* ^2^). Limits of detection (LODs) and quantification (LOQs) were determined based on signal-to-noise ratios (S/N) of 3 and 10, respectively. Reproducibility assessments (five replicate injections) were implemented to ensure data accuracy and reliability.

## Results and Discussion

### Soft and Broad-Spectrum Ionization of the Method

Low ISF is highly desirable for accurate quantitation of PFASs that are highly diverse in species and chemical properties. Our DBDI-HRMS platform demonstrated enhanced analytical performance for ultrashort- and short-chain PFAS analysis, showing superior ionization efficiency and significantly reduced ISF compared to conventional LC-ESI-MS methodologies.^27^ Figure 3 illustrates the precursor and fragment ion profiles of various PFAS subclasses in negative ion mode. The ionization mechanism predominantly generated deprotonated molecular ions [M – H]^−^ as primary precursor ions, with secondary formation of nitrate adducts [M + NO_3_]^−^ and dimeric species [2M – H]^−^. A distinct chain-length dependency was observed in PFCAs, where shorter-chain congeners preferentially formed nitrate adducts, and longer-chain analogues produced more dimeric ions. This structural dependence extended to other PFAS classes, including fluorinated sulfonamides, GenX, and EtFOSAA. Similarly, these exclusively yielded molecular ions and nitrate adducts, with the relative abundance of nitrate increasing proportionally with the chain length. Moreover, the intensity of [M – H–CO_2_]^−^ ions generated in the ion source varies among different PFCA species, with shorter-chain congeners exhibiting a stronger tendency to undergo CO_2_ loss compared to their longer-chain counterparts.

**Figure 3:**
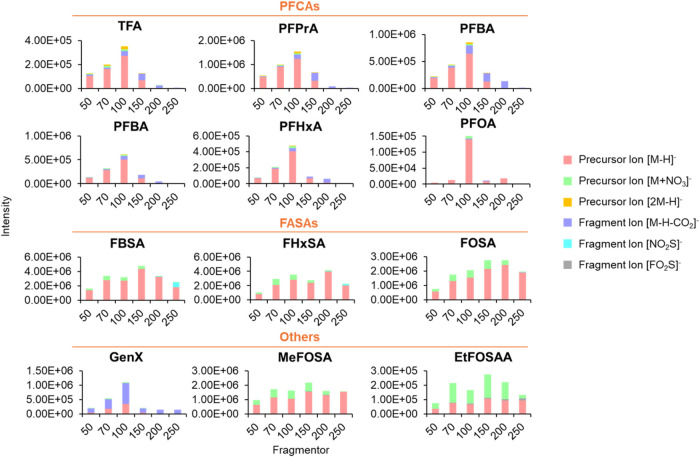
Ionization profiles of PFAS of different subclasses (PFCAs, FASAs, and others). Precursor and fragment ion distributions were derived from high-resolution mass spectra acquired in negative ion mode, with fragmentor voltages optimized between 50 and 250 V.

The fragmentation behavior exhibited a pronounced structural dependence among the PFAS analyzed (Figure 3). GenX, characterized by the labile ether linkages typical of perfluoroether carboxylic acids (PFECAs), demonstrated the highest ISF propensity due to its low bond dissociation energy. In contrast, neutral PFAS such as FASAs showed exceptional stability with minimal fragmentation. Systematic optimization of MS parameters maintained ISF below 20% for all analytes except GenX, even for the ultrashort-chain TFA (Figure S2 and Table S3). Therefore, quantification utilized the [M – H–CO_2_]^−^ fragment for GenX and molecular ions [M – H]^−^ for other PFAS, representing a marked improvement over previously reported LC-ESI-MS methods where ISF exceeded 60% for short-chain PFCAs (PFPrA).^27^ The reduced fragmentation observed with DBDI, attributable to the softer ionization characteristics of plasma-based techniques compared to electrospray processes, significantly enhances spectral interpretability and facilitates reliable precursor ion identification in complex environmental matrices.

Our results demonstrated the utility of the DBDI source in ionizing diverse chemical classes through multiple mechanisms. As illustrated in Table S4, PAEs and OPEs predominantly formed protonated ions [M + H]^+^, while PAHs generated stable molecular ions [M]^+^ through charge transfer. This broad ionization capability extends to a wide range of chemical categories, including aromatic, fluorinated, chlorinated, and oxygen-containing compounds, covering both polar and nonpolar species. The unique plasma chemistry of SICRIT, generating a rich mixture of reactive species (H_3_O^+^, N_2_^+^, and NO^+^ in positive mode, NO_2_^–^, O_2_^–^, and OH^–^ in negative mode), enables comprehensive screening of ultrashort-chain PFAS and copollutants in atmospheric particulates.

### MS/MS Fragmentation and Dopant Effects

Furthermore, we developed an innovative pseudo-MRM approach utilizing targeted MS/MS acquisition, combining advanced data processing algorithms with improved selectivity and accuracy. Moreover, the effect of dopant gas on fragmentation pathways was investigated by using different makeup gases: dry nitrogen, humid nitrogen, and room air. The results showed that the MS/MS fragmentation patterns of most PFAS largely aligned with their observed in-source fragmentation behavior. All analyzed PFCAs exhibited decarboxylation as the dominant fragmentation pathway, characterized by a neutral loss of CO_2_ (44 Da) to form [M – H–CO_2_]^−^ fragment ions, while FASAs consistently produced characteristic [NO_2_S]^−^.

Dopant selection significantly influenced the fragmentation pathways for specific PFAS classes (Figure 4). Notably, humid nitrogen dramatically enhanced the formation of trifluoroacetic acid fragment ions from PFPeA (C5), likely due to water-vapor-mediated fragmentation in the collision cell. A similar, though less pronounced, effect was observed for fluorinated sulfonamides, where humid nitrogen promoted the generation of shorter-chain PFCA fragments. Based on recent studies demonstrating the unexpected activation of inert N_2_ into reactive N_2_O at microdroplet interfaces, we hypothesize that PFAS may similarly undergo unique interfacial transformations—potentially involving water-mediated radical reactions or defluorination pathways.^42^ However, the underlying mechanistic reaction pathways for dopant-dependent fragmentation, potentially tied to the unique plasma dynamics of the DBDI source, remain to be explored.

**Figure 4:**
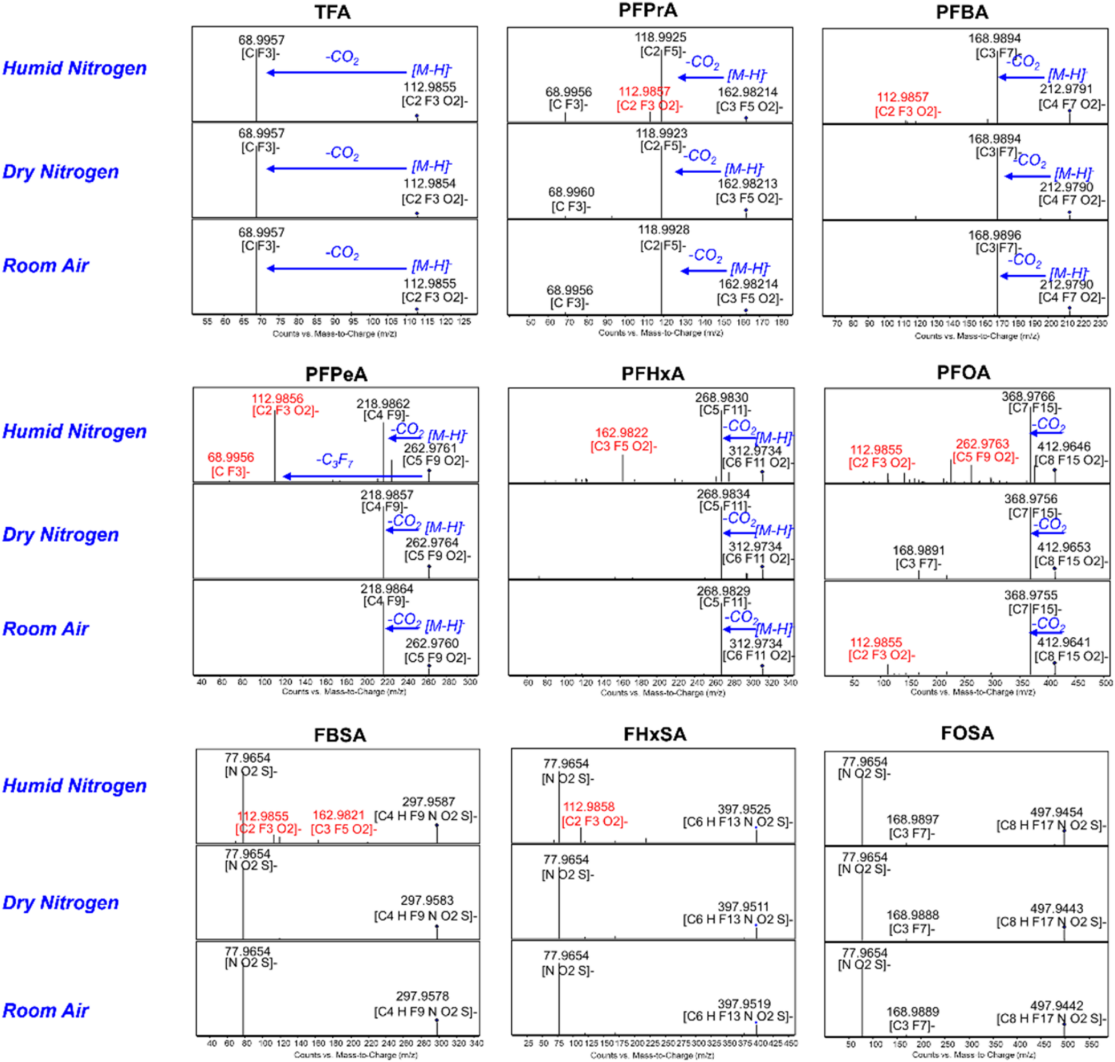
Influence of dopant gas (humid nitrogen, dry nitrogen, and room air) on PFAS fragmentation patterns. Spectra were acquired using a Q-TOF mass spectrometer operated in targeted MS/MS mode. Measured *m*/*z* values, proposed ion formulas, and fragmentation pathways are annotated.

As detailed in Table S5, all measured precursor and quantifier fragment ions demonstrated exceptional mass accuracy with errors consistently below 5 ppm. The optimized parameters for MS/MS acquisition, including pseudo-MRM transitions, fragmentor voltages, and collision energies for all compound classes, are summarized in Table S4.

### Optimization of SPME Conditions

SPME coupled with DBDI-HRMS has emerged as a powerful technique for high-throughput screening of various contaminants, ranging from nonpolar PAHs to polar organic compounds. Based on this, we refined the SPME method for the analysis of PFAS and cooccurring pollutants in complex PM_2.5_ matrices. Method optimization focused on key SPME parameters, including fiber coating selection, extraction temperature, extraction time, and desorption temperature. Initial evaluation of a 65 μm DVB/PDMS fiber revealed selective adsorption of FASAs but inadequate performance for PFCAs—a major PFAS subclass. Comparative assessment of alternative coatings, including 50/30 μm DVB/CAR/PDMS and 85 μm PA, demonstrated significantly enhanced PFCAs recovery efficiencies compared to those of 100 μm PDMS fibers. The 85 μm PA fiber was ultimately selected for its superior and balanced extraction efficiency across all PFAS subclasses, including challenging ultrashort-chain congeners, while maintaining compatibility with the complex PM_2.5_ matrix. Optimal extraction conditions were established at 60 °C for 40 min, followed by desorption at 240 °C, balancing extraction efficiency and thermal stability of sensitive PFAS species (Figure S3 and Table S6).

For simultaneous analysis of PAEs, OPEs, and PAHs, a 65 μm DVB/PDMS fiber was selected based on its bipolar characteristics and optimal mass range (50–350 *m*/*z*). The elevated extraction temperature (100 °C) was essential for the efficient release of high-boiling-point PAHs from the PM_2.5_ matrix, while the moderate desorption temperature (230 °C) was carefully selected to prevent thermal degradation of phthalates, which are prone to in-source fragmentation at higher temperatures. Detailed information on optimized SPME conditions for all classes of compounds is provided in Table S6.

### Method Validation and Identification of PFAS

Method validation for quantitative analysis yielded excellent linearity (*R* ^2^ > 0.995) across all target PFAS analytes (Table S7). Reproducibility was evaluated through triplicate analysis of TFA standards, demonstrating consistent EIC profiles and MS/MS spectral patterns with an RSD of 3% in peak area measurements (Figure S4). Our method achieved exceptional sensitivity, with LODs ranging from 0.06 to 2.02 pg/m^3^ and LOQs between 0.2 and 6.72 pg/m^3^ (Table S7). FASAs exhibited superior sensitivity compared with ionic PFCAs, highlighting the unique ionization characteristics of DBDI for neutral PFAS species.

As a result, application of the optimized SPME-DBDI-MS/MS method to PM_2.5_ filter extracts successfully identified various ultrashort-chain (C2–C3) and short-chain (C4–C6) PFAS in atmospheric particulates. The detection of TFA was confirmed through comparative analysis of extracted ion chromatograms (*m*/*z* 113 → 69) and characteristic MS/MS spectra across blank samples, analytical standards, and environmental PM_2.5_ extracts (Figure 5). The solvent-free nature of our SPME-based approach effectively eliminated background contamination from PFAS present in analytical reagents, as demonstrated by the absence of TFA signals in sample blanks (Figure 5).

**Figure 5:**
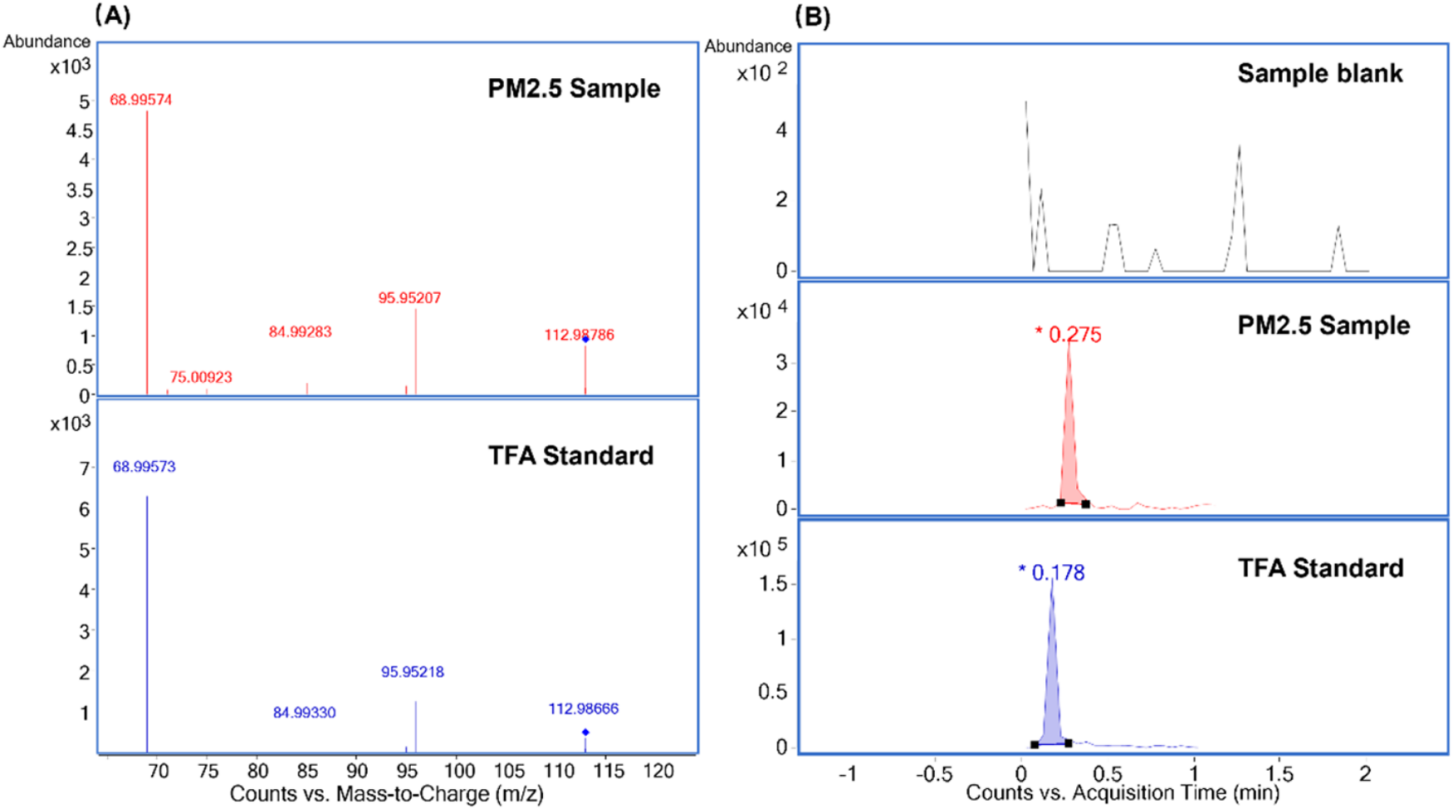
MS/MS spectrum of TFA detected in a PM_2.5_ sample and chemical standard spiked on blank sample (A) and EICs for TFA with the transition *m*/*z* 113 → 69 from a blank sample, a representative PM_2.5_ sample, and the chemical standard (B). Due to the manual injection approach, the retention time may vary among samples, whereas it will not influence the identification of targeted analytes.

### Environmental Concentrations and Seasonal Variations of PFAS in PM_2.5_

As a proof of concept, we applied the developed method to PM_2.5_ samples, a complex environmental matrix containing numerous airborne contaminants.^37–41^ Analysis of PM_2.5_ samples collected in Zhengzhou from December 2023 to August 2024 revealed the ubiquitous presence of PFAS, with distinct phase partitioning and seasonal variations. PFCAs dominated the PFAS profiles, which aligns well with findings from a previous study,^43^ while TFA, the ultrashort-chain analogue, contributes to >90% of total concentrations of PFCAs (Table S8). A distinct seasonal trend was observed for PFAS concentrations, with higher levels measured during the spring and summer seasons (average concentrations of 145.12 and 139.71 pg/m^3^, respectively) compared to the winter season (average concentration of 68.45 pg/m^3^) (Figure 6). Interestingly, the seasonal variation in TFA concentrations appeared to positively correlate with FASAs concentrations and, to a lesser extent, with other PFCAs concentrations, suggesting potential atmospheric transformation pathways where FASAs may serve as precursors for TFA formation through oxidative degradation processes.

**Figure 6:**
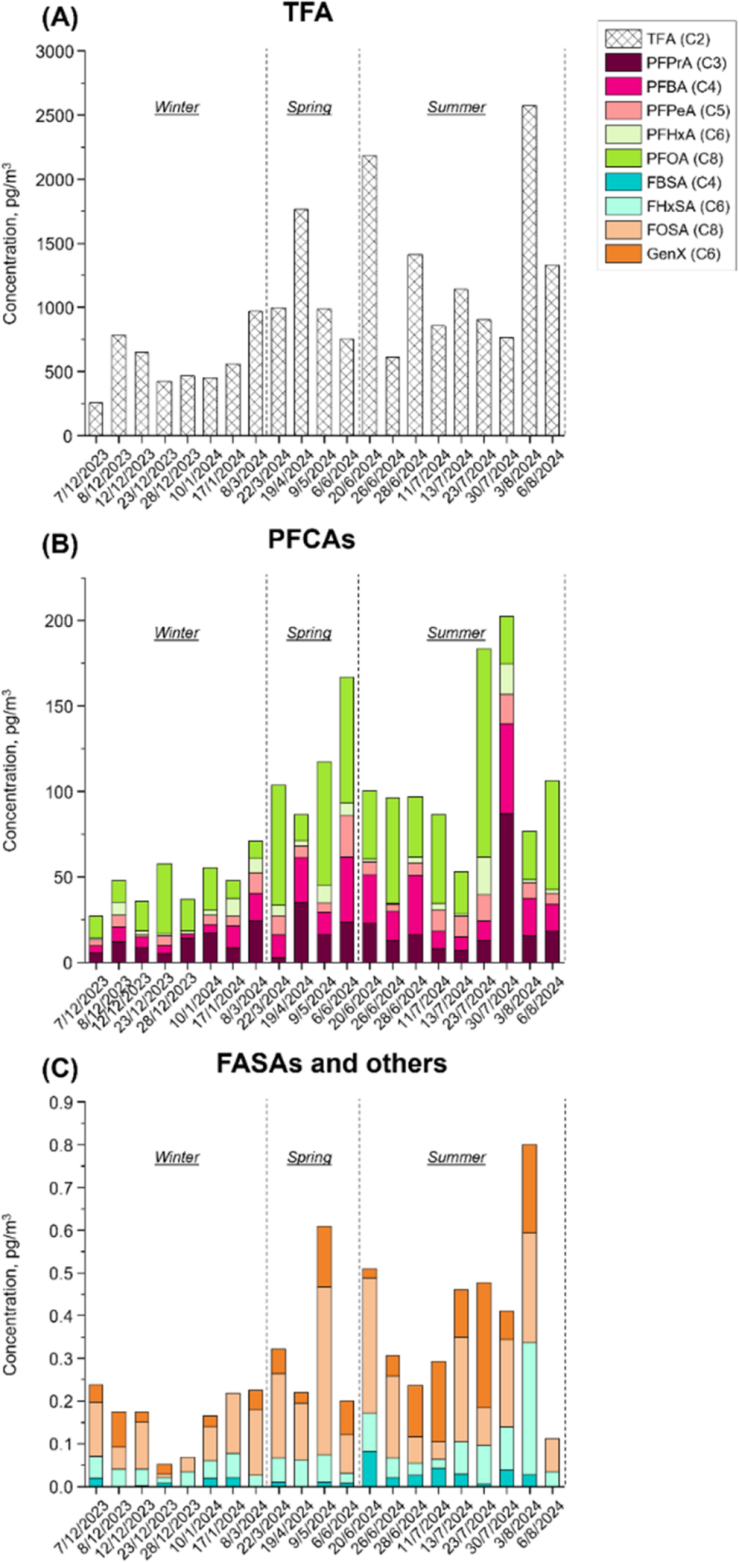
Seasonal variations of the concentrations of TFA (A), PFCAs (B), FASAs, and others (C) detected in PM_2.5_ collected in Zhengzhou from December 2023 to August 2024.

Spearman rank correlation analysis revealed distinct interspecies relationships and potential source signatures (Figure S5 and Table S9). Short-chain PFCAs exhibited positive correlations (0.33 < ρ < 0.78), most notably between PFPrA (C3) and PFBA (C4) (ρ = 0.78). In contrast, TFA showed preferential association with FASAs (TFA-FHxSA: ρ = 0.68), suggesting potential atmospheric transformation from neutral precursors to TFA as the terminal transformation product. GenX demonstrated moderate correlations with both PFOA (ρ = 0.54) and PFHxA (ρ = 0.49), but weaker interspecies relationships among other PFAS classes, suggesting diverse sources and atmospheric processing pathways for different PFAS classes.

### Correlations between PFAS and Cooccurring Pollutants

PM_2.5_ serves as an important transport medium of ambient PFAS and also carries a wide range of cooccurring emerging contaminants, including endocrine-disrupting chemicals, such as PAEs and OPEs, as well as legacy contaminants like PAHs. PAHs, particularly high-molecular-weight congeners like benzo­[*a*]­pyrene, are established carcinogens that induce DNA damage through reactive metabolic intermediates.^44^ PAEs could disrupt endocrine function by interfering with hormone receptors,^45^ while OPEs demonstrate neurodevelopmental toxicity effects.^46^ Moreover, the cooccurrence of multiple classes of contaminants raises concerns regarding potential synergistic toxicity. Associations between PFAS with PAEs and PAHs have been investigated in toxicological studies,^44,47^ while their environmental relationships remain critical for elucidating their sources, combined environmental behavior, and cumulative health impacts. Beyond PFAS, the scope of our study was broadened to encompass additional chemical classes of emerging environmental and health concerns, including PAHs, PAEs, and OPEs. Distinct seasonal trends were observed for different pollutant classes (Figures S6 and S7). PAHs exhibited over ten times elevated concentrations during the winter sampling period compared to spring and summer, consistent with increased emissions from combustion-related activities for heating during colder months. Conversely, PAEs, OPEs, and PFAS displayed higher concentrations in spring and summer, potentially due to a combination of factors such as enhanced volatilization at higher temperatures, increased agricultural applications (for certain OPEs), and elevated product usage during warmer periods. These observed seasonal patterns were consistent with established temporal trends reported in previous atmospheric studies of these pollutant classes,^48–50^ demonstrating the feasibility and reliability of our newly developed approach for characterization of multiple classes of contaminants in PM_2.5_.

A particularly intriguing seasonal-dependent relationship emerged between PFAS and PAEs (Figure S6). A positive correlation emerged during the winter, suggesting common source emissions or shared influencing factors during colder months. However, the relationship underwent a complete reversal in summer, exhibiting a negative correlation. This seasonal inversion likely reflects differential responses to meteorological parameters and atmospheric processes: temperature-dependent partitioning between gas and particulate phases, varying degradation rates under different photochemical conditions, and shifts in dominant emission sources between seasons. The winter correlation may indicate shared indoor sources, while the summer pattern could result from outdoor-dominated processes where PAEs preferentially partition to the gas phase, while PFAS remain particle-bound.

The cooccurrence of multiple classes of contaminants raises concerns regarding potential synergistic toxicity. Previous studies have demonstrated altered phase I biotransformation enzymes in rainbow trout following coexposure to PFAS and benzo­[*a*]­pyrene,^44^ while PFAS–PAEs mixtures have been linked to thyroid disruption in adolescents.^47^ These findings underscore the importance of evaluating contaminant interactions, in addition to individual concentrations. Notably, this study offers an advanced approach that facilitates future investigations into the influence of meteorological factors on cooccurrence patterns and cumulative health impacts.

## Conclusions

This study presents a simple, robust, and solvent-free SPME-DBDI-HRMS/MS method that achieves, for the first time, simultaneous quantification of PFAS covering ultrashort- to long-chain PFAS in a single run. The method demonstrated reduced ISF compared to the conventional LC-ESI-MS method over 60% while maintaining broad-spectrum capability across diverse PFAS classes. By removing solvent usage to minimize background, this sustainable method achieved exceptional sensitivity of 0.06–2.02 pg/m^3^ for comprehensive PFAS analysis. Compared with DART-MS, SICRIT offers superior ionization efficiency for ultrashort-chain PFAS through its extended MS inlet design, which enhances ion–molecule interactions. While no prior studies have successfully detected ultrashort-chain analogues using ambient ionization MS, direct performance comparisons are currently limited by this data gap. As a proof of concept, results from PM_2.5_ samples collected in the representative city revealed the ubiquitous presence of PFAS, predominantly the ultrashort-chain PFCAs. Furthermore, we demonstrated the expandable utility of the method by analyzing other cooccurring environmental pollutants including PAHs, PAEs, and OPEs, providing valuable insights into their temporal correlations with PFAS. Our method consequently enables future environmental mechanistic studies to inform regulatory policies and enhance environmental monitoring strategies.

## Supplementary Material



## Notes

Collection dates of PM_2.5_ samples during December 2023–August 2024; optimized MS parameters for positive and negative ionization modes, including skimmer and capillary voltages and source temperatures; in-source fragmentation ratios of PFAS analyzed by DBDI-HRMS; optimized PRM transitions, fragmentors, and collision energies optimized for all classes of compounds; precursor and product ions for PFAS analyzed using targeted MS/MS acquisition; optimized SPME conditions for PAEs, OPEs, and PAHs; calibration parameters and reproducibility for individual PFAS, PAEs, OPEs, and PAHs; concentrations (pg/m^3^) of PFAS detected in PM_2.5_ samples collected from Zhengzhou; correlation analysis (coefficient values) of individual PFAS measured in PM_2.5_ samples; molecular structures of PAEs, OPEs, and PAHs analyzed in this study; in-source fragmentation for TFA and other PFAS; effects of SPME conditions on PFAS analysis, including the desorption temperature, extraction duration, and SPME fiber materials; typical EICs and MS/MS spectra obtained from three repeated injections of the same concentrations of TFA using SPME-DBDI-HRMS/MS; spearman rank correlation analysis of interspecies relationships among PFAS detected in PM_2.5_; temporal trends observed for different classes of pollutants; and seasonal variations of the concentrations of PAHs, PAEs, and OPEs detected in PM_2.5_ collected in Zhengzhou from December 2023 to August 2024 (PDF)
