# Supplementary material for: A Comprehensive, Simple, Robust, and Solvent-Free Method Covering Ultrashort- to Long-Chain PFAS in Atmospheric Samples
Source: Anal Chem. 2025 Jul 2;97(27):14838–46. doi: 10.1021/acs.analchem.5c03123 (PMC12268817; doi:10.1021/acs.analchem.5c03123)
Supplement: Supplementary file 1 [file ac5c03123_si_001.pdf]

SUPPORTING INFORMATION

A comprehensive, simple, robust, and solvent-free method covering ultrashort- to long-chain PFAS in atmospheric samples

Wanlin Guo,[a] Yanhao Zhang,[a,b] Yawei Wang,[c] Lin Zhu,[a] Zongwei Cai [a,d*]*

[a] State Key Laboratory of Environmental and Biological Analysis, Department of Chemistry, Hong Kong Baptist University, Hong Kong SAR, 999077, China

[b] School of Ecology and Environment, Zhengzhou University, Zhengzhou, 450001, China

[c] State Key Laboratory of Environmental Chemistry and Ecotoxicology, Research Center for Eco-Environmental Sciences, Chinese Academy of Sciences, Beijing, 100085, China

[d] Eastern Institute of Technology, Ningbo, 315200, China

* Corresponding authors: zhu_lin@hkbu.edu.hk, zwcai@hkbu.edu.hk

Table of Contents

Table S1. Collection dates of the $PM_{2.5}$ samples during December 2023 to August 2024.

Table S2. Optimized MS parameters for positive and negative ionization modes, including skimmer and capillary voltages and source temperatures.

Table S3. The in-source fragmentation ratios of PFAS analyzed by DBDI-HRMS.

Table S4. The optimized polarities, precursor ion species, PRM transitions, fragmentors, and collision energies optimized for all classes of compounds.

Table S5. Precursor and product ions for PFAS analyzed using targeted MS/MS acquisition.

Table S6. Optimized SPME conditions for PAEs, OPEs, and PAHs.

Table S7. Calibration parameter and reproducibility for individual PFAS, PAEs, OPEs, and PAHs.

Table S8. Concentrations (pg/m^3) of PFAS detected in $PM_{2.5}$ samples collected from Zhengzhou.

Table S9. Correlation analysis (coefficient values) of individual PFAS measured in $PM_{2.5}$ samples.

Figure S1. Molecular structure of PAEs, OPEs, and PAHs analyzed in this study.

Figure S2. In-source fragmentation for TFA and other PFAS.

Figure S3. The effects of SPME conditions on PFAS analysis, including desorption temperature, extraction duration, desorption temperature, and SPME fiber materials.

Figure S4. Typical EICs and MS/MS spectrums obtained from three repeated injections of the same concentrations of TFA using SPME-DBDI-HRMS/MS.

Figure S5. Spearman rank correlation analysis of interspecies relationships among PFAS detected in $PM_{2.5}$.

Figure S6. Temporal trends observed for different classes of pollutants.

Figure S7. Seasonal variations of the concentrations of PAHs, PAEs, and OPEs detected in $PM_{2.5}$ collected in Zhengzhou from December 2023 to August 2024.

Table S1. Collection dates of the $PM_{2.5}$ samples during December 2023 to August 2024.

**Date**	**Season**
7/12/2023	Winter
8/12/2023	Winter
12/12/2023	Winter
23/12/2023	Winter
28/12/2023	Winter
10/1/2024	Winter
17/1/2024	Winter
8/3/2024	Winter
22/3/2024	Spring
19/4/2024	Spring
9/5/2024	Spring
6/6/2024	Spring
20/6/2024	Summer
26/6/2024	Summer
28/6/2024	Summer
11/7/2024	Summer
13/7/2024	Summer
23/7/2024	Summer
30/7/2024	Summer
3/8/2024	Summer
6/8/2024	Summer

Table S2. Optimized MS parameters for positive and negative ionization modes, including skimmer and capillary voltages and source temperatures.

	Positive	Negative
Gas temp (°C)	320	200
Drying gas (l/min)	8	8
Nebulizer (psi)	6	6
Capillary voltage (V)	10	10
Octopole RF (V)	750	750
Skimmer (V)	60	60
Reference mass	149.023326	124.984009
Iso width (*m/z*)	1.3	1.3
MS acquisition mass range (*m/z*)	65-1000	65-1000
MS acquisition rate (spectra/s)	3	3
MS acquisition time (ms/spectrum)	333.33	333.33
MS/MS acquisition mass range (*m/z*)	65-1000	65-1000
MS/MS acquisition rate (spectra/s)	6	6
MS/MS acquisition time (ms/spectrum)	166.67	166.67

Table S3. The in-source fragmentation ratios of PFAS analyzed by DBDI-HRMS.

	Frag, %
TFA	13%
PFPrA	13%
PFBA	19%
PFPeA	12%
PFHxA	8%
PFOA	2%
FBSA	0%
FHxSA	0%
FOSA	0%
GenX	68%
MeFOSA	1%
EtFOSAA	6%

Table S4. The optimized polarities, precursor ion species, PRM transitions, fragmentors, and collision energies optimized for all classes of compounds.

Class	Compound name	Compound Formula	Polarity	Precursor Ion Specie	PRM Transition	Fragmentor (V)	CE (V)
PFAS	TFA	C2HF3O2	Negative	$[M-H]^-$	113>69	100	10
PFAS	PFPrA	C3HF5O2	Negative	$[M-H]^-$	163>119	100	10
PFAS	PFBA	C4HF7O2	Negative	$[M-H]^-$	213>169	100	5
PFAS	PFPeA	C5HF9O2	Negative	$[M-H]^-$	263>219	100	5
PFAS	PFHxA	C6HF11O2	Negative	$[M-H]^-$	313>269	100	5
PFAS	PFOA	C8HF15O2	Negative	$[M-H]^-$	413>369	100	5
PFAS	FBSA	C4F9SO2NH2	Negative	$[M-H]^-$	298>78	150	20
PFAS	FHxSA	C6F13SO2NH2	Negative	$[M-H]^-$	398>78	200	25
PFAS	FOSA	C8F17SO2NH2	Negative	$[M-H]^-$	498>78	250	30
PFAS	GenX	C6HF11O3	Negative	$[M-H]^-$	285>169	100	5
PFAS	MeFOSA	C9H4F17NO2S	Negative	$[M-H]^-$	512>169	150	35
PFAS	EtFOSAA	C12H8F17NO4S	Negative	$[M-H]^-$	584>419	100	5
PAEs	DMP	C10H10O4	Positive	$[M+H]^+$	195>163	175	15
PAEs	DEP	C12H14O4	Positive	$[M+H]^+$	223>207	175	8
PAEs	DBP	C16H22O4	Positive	$[M+H]^+$	279>149	175	5
PAEs	BBP	C19H20O4	Positive	$[M+H]^+$	313>91	175	5
PAEs	DEHA	C22H42O4	Positive	$[M+H]^+$	371>355	300	8
PAEs	DNOP	C24H38O4	Positive	$[M+H]^+$	391>149	250	5
OPEs	TCEP	C6H12Cl3O4P	Positive	$[M+H]^+$	285>269	250	20
OPEs	TCPP	C9H18Cl3O4P	Positive	$[M+H]^+$	327>215	250	30
OPEs	TDCPP	C9H15Cl6O4P	Positive	$[M+H]^+$	431>415	300	25
PAHs	ACY	C12H8	Positive	$[M]^+$	152>150	250	40
PAHs	ACE	C12H10	Positive	$[M]^+$	154>152	250	30
PAHs	PHE/ANT	C14H10	Positive	$[M]^+$	178>152	250	40
PAHs	FLE	C13H10	Positive	$[M]^+$	166>165	250	30
PAHs	PYR/FLA	C16H10	Positive	$[M]^+$	202>200	200	60

Table S5. Precursor and product ions for PFAS analyzed using targeted MS/MS acquisition.

Compound name	Formula	Precursor Ion Specie	Measured *m/z*[a]	Diff (ppm)[a]	Product Ion Specie	Measured *m/z*[b]	Diff (ppm)[b]
TFA	$C_2HF_3O_2$	$[M-H]^-$	112.9858	1.78	$[M-H-CO_2]^-$	68.9957	-1.38
PFPrA	$C_3HF_5O_2$	$[M-H]^-$	162.9830	3.64	$[M-H-CO_2]^-$	118.9924	-1.1
PFBA	$C_4HF_7O_2$	$[M-H]^-$	212.9800	3.77	$[M-H-CO_2]^-$	168.9895	1.01
PFPeA	$C_5HF_9O_2$	$[M-H]^-$	262.9765	1.95	$[M-H-CO_2]^-$	218.9855	-3.27
PFHxA	$C_6HF_{11}O_2$	$[M-H]^-$	312.9731	0.92	$[M-H-CO_2]^-$	268.9834	1.52
PFOA	$C_8HF_{15}O_2$	$[M-H]^-$	412.9656	-2.05	$[M-H-CO_2]^-$	368.9759	-1.99
FBSA	$C_4F_9SO_2NH_2$	$[M-H]^-$	297.9592	0.83	$[NO_2S]^-$	77.9655	-0.78
FHxSA	$C_6F_{13}SO_2NH_2$	$[M-H]^-$	397.9522	-0.88	$[NO_2S]^-$	77.9653	-0.88
FOSA	$C_8F_{17}SO_2NH_2$	$[M-H]^-$	497.9460	-0.47	$[NO_2S]^-$	77.9655	0.98
GenX	$C_6HF_{11}O_3$	$[M-H-CO_2]^-$	284.9779	0.08	$[C_3F_7]^-$	168.9895	0.17
MeFOSA	$C_9H_4F_{17}NO_2S$	$[M-H]^-$	511.9600	-3.54	$[C_3F_7]^-$	168.9898	2.25
EtFOSAA	$C_{12}H_8F_{17}NO_4S$	$[M-H]^-$	583.9825	-0.86	$[C_8F_{17}]^-$	418.9730	-0.93

[a] Measured *m/z* and mass difference of precursor ions

[b] Measured *m/z* and mass difference of product ions

Table S6. Optimized SPME conditions for PAEs, OPEs, and PAHs.

	PFAS	PAEs/PAHs/OPEs
SPME fiber	PA	DVB/PDMS
Extraction temperature, ℃	60	100
Extraction duration, min	40	40
Desorption temperature, ℃	240	230

Table S7. Calibration parameter and reproducibility for individual PFAS, PAEs, OPEs, and PAHs.

	R2	LOD, pg/m3	LOQ, pg/m3
TFA	0.996	0.19	0.64
PFPrA	0.997	0.18	0.58
PFBA	0.9955	0.77	2.58
PFPeA	0.9962	2.02	6.72
PFHxA	0.9958	1.69	5.65
PFOA	0.9965	6.97	23.25
FBSA	0.9968	0.06	0.20
FHxSA	0.9959	0.14	0.47
FOSA	0.9961	0.23	0.76
GenX	0.9963	0.16	0.53
MeFOSA	0.996	0.15	0.49
EtFOSAA	0.9957	0.32	1.08
DMP	0.9964	0.04	0.14
DEP	0.9956	0.03	0.10
DBP	0.9962	0.05	0.16
BBP	0.9959	0.88	2.95
DEHA	0.9961	0.36	1.22
DNOP	0.9958	3.44	11.48
TCEP	0.9957	3.95	13.17
TCPP	0.996	1.39	4.64
TDCPP	0.9956	0.55	1.83
ACY	0.9958	0.76	2.53
ACE	0.9959	1.08	3.61
PHE/ANT	0.996	0.41	1.38
FLE	0.9957	0.65	2.15
PYR/FLA	0.9958	0.82	2.73

Table S8. Concentrations (pg/m^3) of PFAS detected in $PM_{2.5}$ samples collected from Zhengzhou.

Date	TFA	PFPrA	PFBA	PFPeA	PFHxA	PFOA	FBSA	FHxSA	FOSA	GenX
7/12/2023	255.84	5.64	3.83	3.27	1.45	12.63	0.02	0.05	0.13	0.04
8/12/2023	783.32	11.91	8.78	6.71	7.74	12.48	NA	0.04	0.05	0.08
12/12/2023	646.57	8.74	5.82	1.34	2.32	17.35	NA	0.04	0.11	0.03
23/12/2023	422.19	5.09	4.55	5.54	1.54	40.74	0.01	0.01	0.01	0.02
28/12/2023	464.60	14.20	2.29	NA	1.81	18.34	NA	0.03	0.03	NA
10/1/2024	450.24	17.34	4.36	5.55	3.18	24.96	0.02	0.04	0.08	0.02
17/1/2024	557.18	8.63	12.60	5.80	10.14	10.42	0.02	0.06	0.14	NA
8/3/2024	969.21	24.12	15.83	12.46	8.35	10.04	NA	0.03	0.15	0.04
22/3/2024	995.95	2.91	13.03	11.10	6.10	70.26	0.01	0.06	0.20	0.06
19/4/2024	1761.93	35.21	26.01	6.47	3.30	15.44	NA	0.06	0.13	0.03
9/5/2024	985.42	15.73	13.58	5.33	10.42	72.05	0.01	0.06	0.39	0.14
6/6/2024	753.56	23.72	37.66	24.02	7.61	73.85	0.01	0.02	0.09	0.08
20/6/2024	2183.25	22.72	28.15	7.92	1.36	40.23	0.08	0.09	0.32	0.02
26/6/2024	611.98	12.29	17.38	4.00	0.87	61.49	0.02	0.05	0.19	0.05
28/6/2024	1409.47	15.76	34.82	7.42	3.60	35.04	0.03	0.03	0.06	0.12
11/7/2024	855.28	7.93	9.97	12.69	3.93	51.83	0.04	0.02	0.04	0.19
13/7/2024	1140.18	6.80	8.02	12.04	1.40	24.73	0.03	0.08	0.25	0.11
23/7/2024	903.11	12.65	11.27	15.62	21.80	122.20	NA	0.09	0.09	0.29
30/7/2024	764.86	86.97	52.19	17.60	17.60	28.19	0.04	0.10	0.20	0.07
3/8/2024	2572.79	15.40	21.62	9.47	1.66	28.57	0.03	0.31	0.26	0.21
6/8/2024	1326.70	18.02	15.81	6.55	2.00	63.74	NA	0.03	0.08	NA

Table S9. Correlation analysis (coefficient values) of individual PFAS measured in $PM_{2.5}$ samples.

	FBSA	FHxSA	FOSA	GenX	TFA	PFPrA	PFBA	PFPeA	PFHxA	PFOA
FBSA	1.0	0.3	0.4	0.0	0.4	0.2	0.4	0.1	-0.1	0.0
FHxSA	0.3	1.0	0.5	0.5	0.7	0.2	0.2	0.1	0.0	0.0
FOSA	0.4	0.5	1.0	0.1	0.5	0.2	0.3	0.0	0.0	0.0
GenX	0.0	0.5	0.1	1.0	0.3	0.0	0.0	0.4	0.5	0.6
TFA	0.4	0.7	0.5	0.3	1.0	0.1	0.4	0.1	-0.2	0.0
PFPrA	0.2	0.2	0.2	0.0	0.1	1.0	0.8	0.4	0.5	-0.1
PFBA	0.4	0.2	0.3	0.0	0.4	0.8	1.0	0.6	0.3	0.1
PFPeA	0.1	0.1	0.0	0.4	0.1	0.4	0.6	1.0	0.5	0.4
PFHxA	-0.1	0.0	0.0	0.5	-0.2	0.5	0.3	0.5	1.0	0.4
PFOA	0.0	0.0	0.0	0.6	0.0	-0.1	0.1	0.4	0.4	1.0

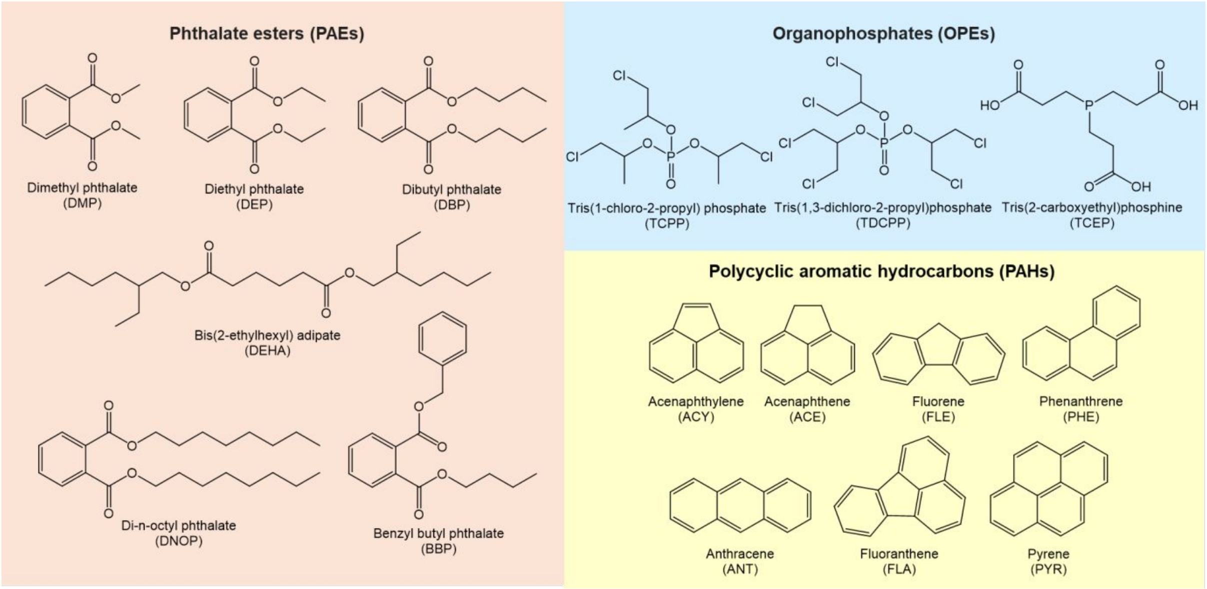

Figure S1. Molecular structure of PAEs, OPEs, and PAHs analyzed in this study. All compounds were monitored simultaneously within a single injection operated in positive mode.

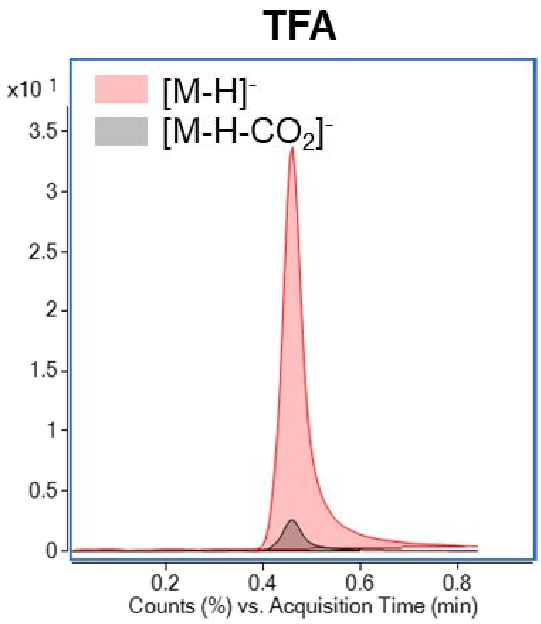

Figure S2. In-source fragmentation for TFA and other PFAS. The ISF ratio was calculated as the peak area of an individual ISF ion divided by the sum of the peak areas of the molecular ion and all ISF ions.

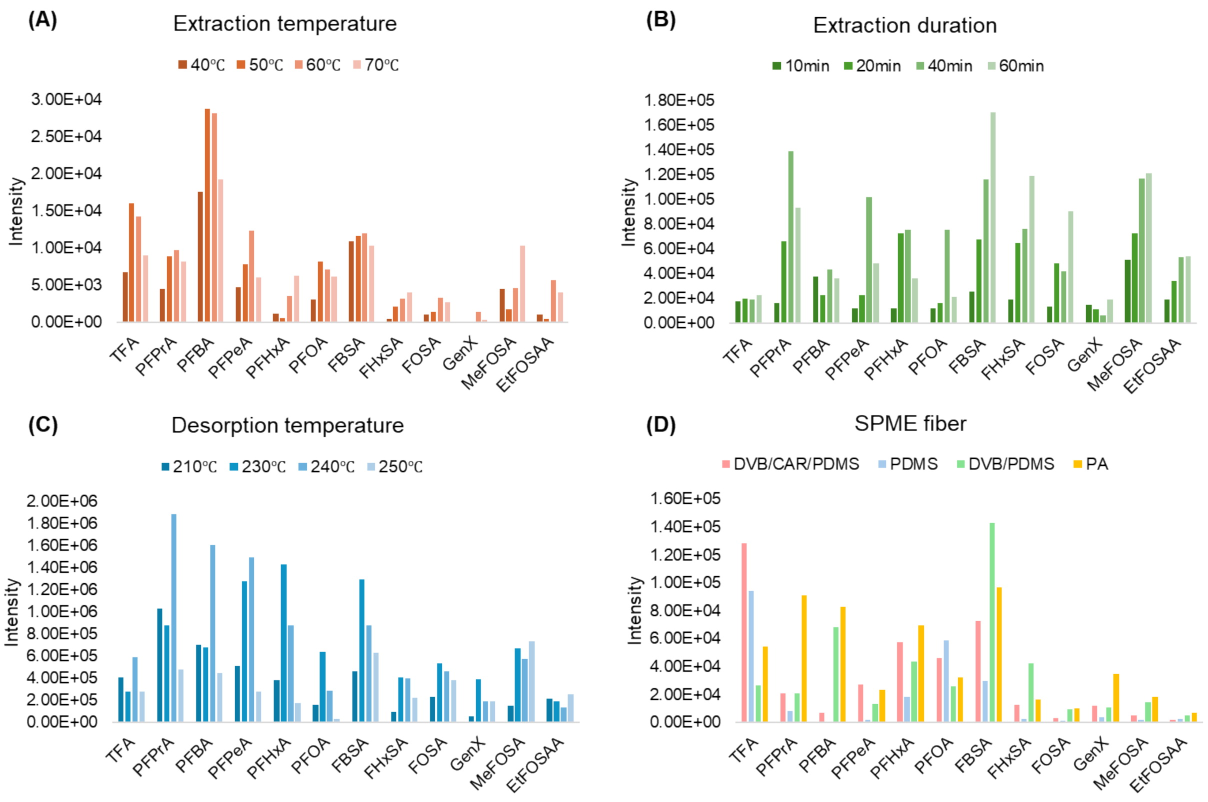

Figure S3. The effects of SPME conditions on PFAS analysis, including desorption temperature, extraction duration, desorption temperature, and SPME fiber materials.

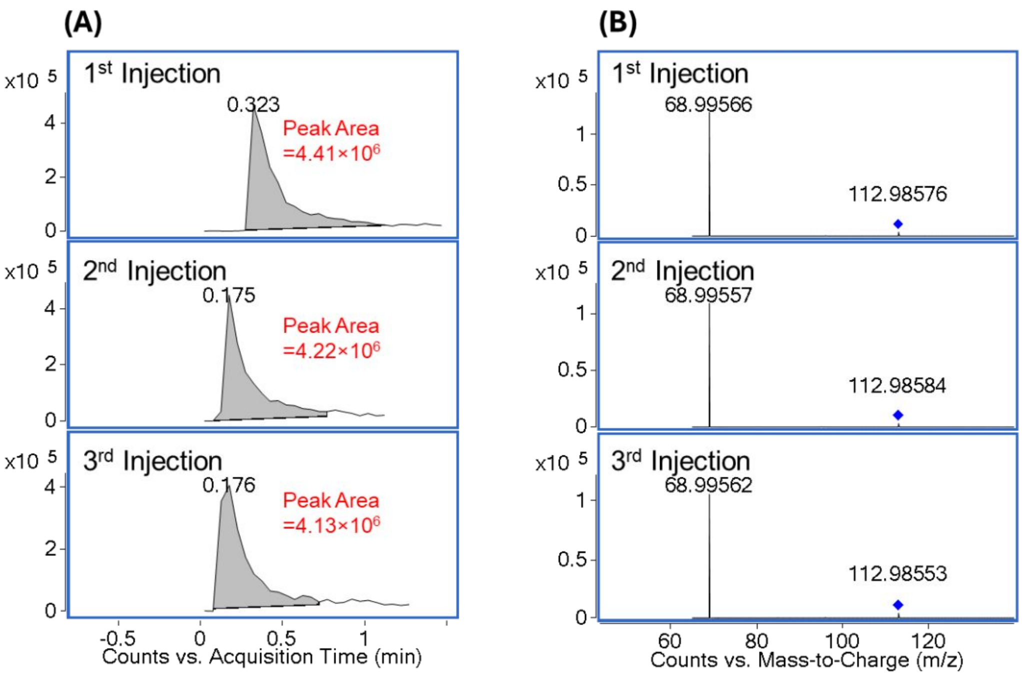

Figure S4. Typical EICs and MS/MS spectrums obtained from three repeated injections of the same concentrations of TFA using SPME-DBDI-HRMS/MS.

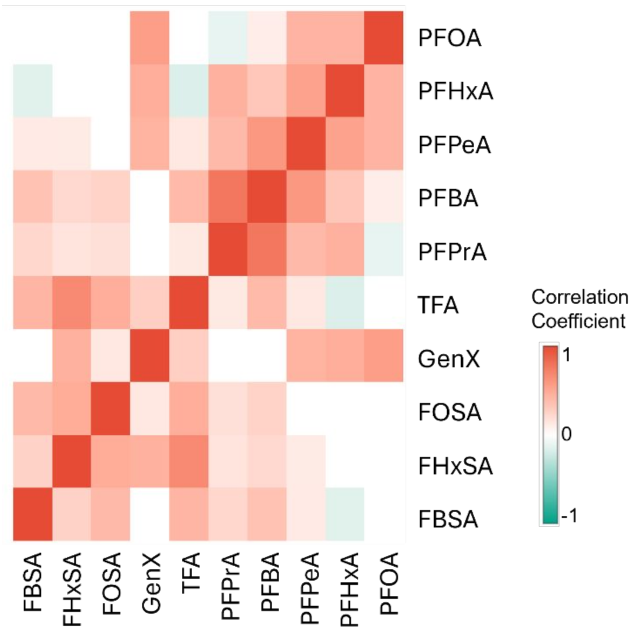

Figure S5. Spearman rank correlation analysis of interspecies relationships among PFAS detected in $PM_{2.5}$.

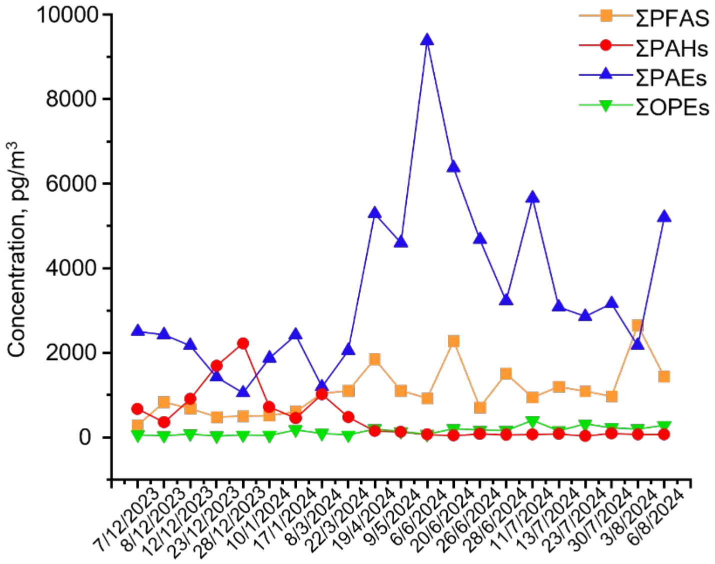

Figure S6. Temporal trends observed for different classes of pollutants.

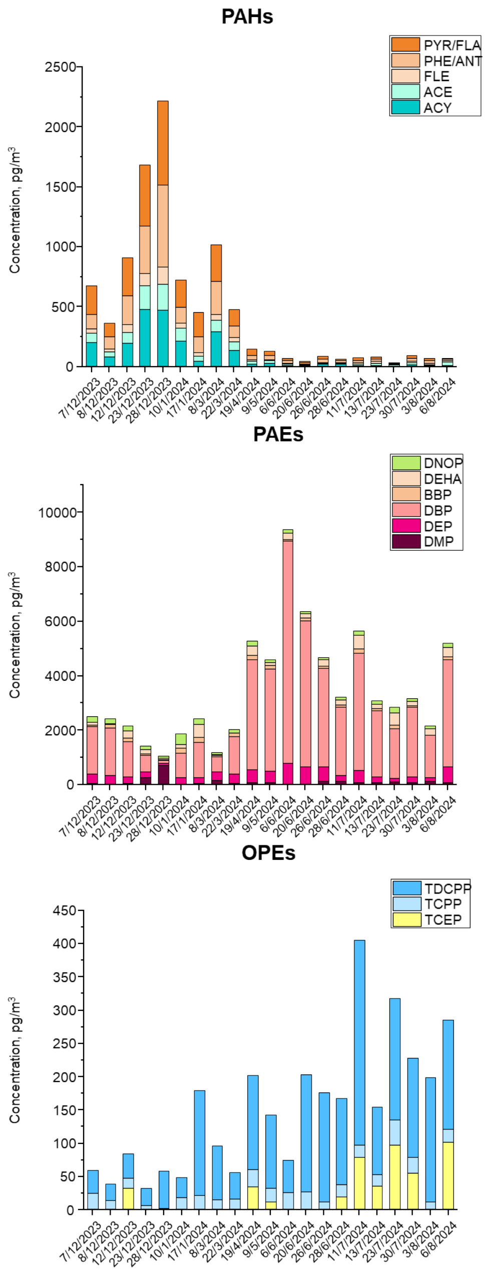

Figure S7. Seasonal variations of the concentrations of PAHs, PAEs, and OPEs detected in $PM_{2.5}$ collected in Zhengzhou from December 2023 to August 2024.
